# Analysis of harmony between color and fragrance in lighting environments by the reaction of the orbitofrontal area

**DOI:** 10.1177/20416695221102191

**Published:** 2022-05-22

**Authors:** Gaku Yamashita, Midori Tanaka, Takahiko Horiuchi

**Affiliations:** Graduate School of Science and Engineering, 12737 Chiba University, Chiba, Japan; Graduate School of Global and Transdisciplinary Studies, 12737 Chiba University, Chiba, Japan; Graduate School of Science and Engineering, 12737 Chiba University, Chiba, Japan

**Keywords:** cross-modal effect, harmony, orbitofrontal area, NIRS

## Abstract

This study focuses on the analysis of the cross-modal effects between sight (color) and smell (fragrance). While most previous researches have studied the harmony of color and fragrance using small-field colors such as patches and display stimuli, this study analyzes harmony in lighting environments. In our experiments, we focused on the finding that emotional states manifest themselves in responses in the orbitofrontal cortex and used near-infrared spectroscopy to evaluate orbitofrontal responses. Five different aromas were prepared for fragmentation. Initially, the observers were asked to select the most pleasant and least unpleasant aromas. The two selected aromas were soaked in non-fat cotton cloth and placed in a light-shielding brown bottle, which was used as a scent stimulus. For the lighting environment, 36 different lighting colors were designed using a luminaire consisting of 14 LEDs. The results of the experiment showed that the lighting color that the observers judged to be harmonious by actually smelling the fragrance (sensory color) activated the orbitofrontal cortex more than the lighting color that they judged to be harmonious by recalling the name of the fragrance (imagery color).

## Introduction

In our daily lives, we obtain information from five senses (sight, hearing, smell, taste, and touch) and make action selections. There is more than one smell modality in the outside world, and simultaneously, there are multisensory modalities; therefore, a comprehensive judgment is made from that information. In recent years, research on the five senses, which has been conducted independently in the past, has been active in multi-smell research referred to as cross-modal or multimodal. This study focused on the cross-modal effects of sight and smell. Smells are often perceived using visual cues. Both sensations interact to modulate the subjective experience of the stimuli from which they emanate.

In the 1990s, Zeller et al. showed that color is a visual sign that affects the perception of smell (Zellner & Kautz, 1990; Zellner & Whitten, 1999). Gilbert et al. proposed the existence of robust correspondence between vision and olfaction (Gilbert et al., 1996). Kemp et al. evaluated aspects of the cross-modal associations between vision and olfaction: whether the perceptual dimensions of odor vary systemically with those of vision (Kemp & Gilbert, 1997). At the turn of the century, Luisa et al. suggested that odor–color associations can be both systematic and robust (Luisa et al., 2006). Saito et al. extracted dimensions in impressions of colors and fragrances and constructed a harmonious color model with fragrances (Miura & Saito, 2012a; Miura & Saito, 2012b). Ainoya et al. demonstrated the relationship between aroma and color and examined the physiological and psychological effects of VDT (Ainoya & Kasamatsu, 2016). In these studies, evaluations were conducted according to the subjectivity of the observers, including scaling and rating (Gilbert et al., 1996; Kemp & Gilbert, 1997; Zellner & Kautz, 1990; Zellner & Whitten, 1999), color matching (Luisa et al., 2006), and impression evaluation (Ainoya & Kasamatsu, 2016; Miura & Saito, 2012a; Miura & Saito, 2012b). However, subjective evaluation by the observer has the problem of adaptation and ambiguity because of the repetition of the experiment. Therefore, there have been attempts to analyze cross-modality by observing human biological reactions (Danja et al., 2019; Gottfried & Dolan, 2003; Osterbauer et al., 2005; Ishimaru et al., 2004; Saito et al., 2012; Sara et al., 2019).

Danja et al. tested the effect of multisensory stimulation on information processing in the human posterior piriform cortex, a region linked to olfactory object encoding (Danja et al., 2019). Robert et al. demonstrated a neurophysiological correlation between these cross-modal visual influences on olfactory perception using functional magnetic resonance imaging (fMRI) (Osterbauer et al., 2005). They showed that activity in the caudal regions of the orbitofrontal cortex and insular cortex increased progressively with perceived congruency of the odor–color pairs. Saito et al. (2012) used fMRI to validate a cross-modal experiment in which observers perceived their favorite fragrance and color. When color and fragrance are judged to be highly congruent, the orbitofrontal cortex is activated, as shown by Osterbauer et al. (2005). Gottfried and Dolan (2003) conducted a cross-modal experiment between scent and an image, and reported significant activation of the orbitofrontal cortex on the left side.

Thus, while cross-modal studies of color and scent have been conducted, the color stimuli they target are narrow-field colors from colored liquids (Zellner & Kautz, 1990; Zellner & Whitten, 1999), colored chips (Gilbert et al., 1996; Kemp & Gilbert, 1997; Miura & Saito, 2012a; Miura & Saito, 2012b), monitor colors (Ainoya & Kasamatsu, 2016; Danja et al., 2019; Gottfried & Dolan, 2003; Luisa et al., 2006; Osterbauer et al., 2005), and video images (Saito et al., 2012). In this study, we analyze harmony as a cross-modal effect between wide-field colors and fragrances in lighting environments. As shown by Osterbauer et al. (2005) and Saito et al. (2012), when color and fragrance were judged to be more harmonious, the orbitofrontal cortex was shown to be activated. Sara et al. (2019) and Ishimaru et al. (2004) also reported results in which stimulus harmony, although an olfactory stimulus, caused the activation of the orbitofrontal cortex. Therefore, we focused on the finding that emotional states manifest in responses in the orbitofrontal cortex. To measure the orbitofrontal responses, we used near-infrared spectroscopy (NIRS). NIRS is a wearable device that can measure oxyHb concentrations; in addition to being easier to perform physical experiments than conventional devices, reproducibility has also been confirmed (Kakimoto et al., 2009; Kono et al., 2007).

### Methods

#### Experimental Stimuli

##### Visual Stimulation

**Figure 1.**
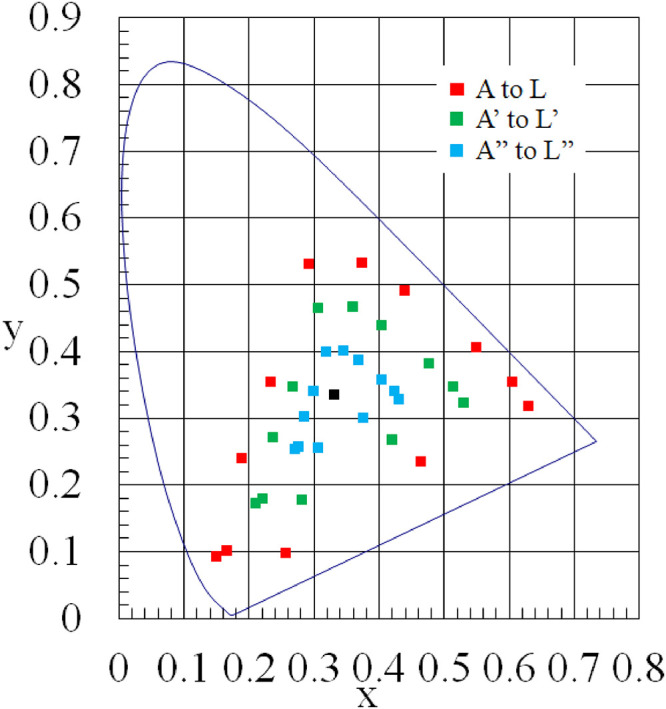
xy chromaticity distribution of lighting colors.

We used a spectrally tunable lighting device (LEDCube, THOUSLITE) for visual stimulation. The LEDCube comprises 14 types of LEDs, and it is possible to design lighting colors with various spectral distributions. In these experiments, we referred to the practical color co-ordinate system (PCCS) when designing lighting. The PCCS is a color system developed by the Japan Color Research Institute with the main purpose of systematically solving the problem of color harmony and can be used as a two-dimensional system based on hue and tone. We selected 12 types of color names defined by PCCS at regular intervals (A: Poppy Red, B: Vermilion, C: Tanjarin, D: Dandelion, E: Fresh Green, F: Emerald Green, G: Peacock Green, H: Cyan, I: Ultra Marine, J: Bellflower, K: Mauve, L: Magenta). We calculated the *x*–*y* values from the RGB values of each color and input them into the LEDCube to reproduce the lighting color. We also considered different lighting saturations for each color to verify a more harmonious cross-modal effect. We set white light (*x*, *y*) = (0.333, 0.333) as the origin, the lighting colors in which each color coordinate is reduced to two-thirds were A' to L', and the lighting colors in which each color was reduced to one-third were A" to L". It was determined that these 36 types of lighting colors would be used for visual stimulation. In these experiments, the illuminance was fixed at 50 lx. The *x*–*y* values of the lighting colors used are shown in Figure 1.

##### Smell Stimulation

In this study, essential oils were used for the smell stimulation. Because essential oils have a relaxing effect on humans, it can be inferred that a physiological response is likely to be obtained. Furthermore, they are considered suitable for this experiment because they emit a small amount of strong scent and it is possible to prepare numerous types.

In conventional studies, when selecting a scent harmony color, the object color itself (e.g., strawberry: red; lemon: yellow) was often the harmony color. However, this method may lead to prejudice and significantly influence the evaluation. Thus, it was considered necessary to verify a cross-modal experiment of more harmonious colors and scents. Therefore, in this study, we compared the cross-modal effect of the lighting color that humans recall when they hear the name of the scent and the lighting color that they feel when they actually smell the scent. In other words, the former is a harmony color selected from the image of the object, as in conventional research. The latter is a color that humans sensuously feel in harmony with its scent. By comparing the cross-modal effects of the two, we inferred that it would lead to the discovery of colors and scents that could not be discovered in the past and that it could be applied in space design.

The essential oil used should be such that the observer can easily recall the harmony color by hearing the name. Therefore, in this experiment, we decided to use five types of essential oils, vanilla, mint, lime, grapefruit, and cypress, which were familiar to the observers.

**Figure 2.**
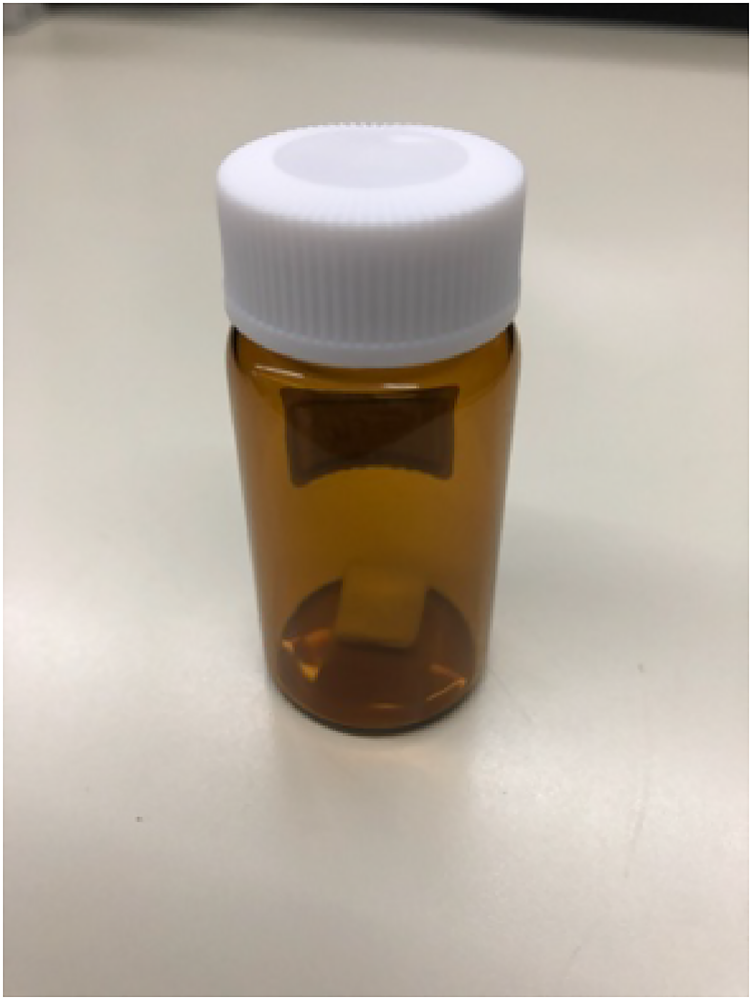
Smell stimulation.

We soaked the essential oil in cotton wool cut into 1 cm squares and placed it in a 20 ml brown shading bottle to stimulate the sense of smell. We then adjusted the intensity of the fragrance to approximately 100 in an odor meter (OMX-SRM, Shinei Technology Co., Ltd.) near the entrance of the brown shading bottle. The smell stimulation used in this study is shown in Figure 2.

#### Experiment

##### Outline

In this experiment, we conducted a cross-modal experiment considering the harmony between the color and scent. As mentioned above, in conventional research using color and scent, we believe that there are two problems that have not yet been verified. First, small-field stimuli, such as color patches, are often used for colors that are visually stimulating, and the cross-modal effect of colors and scents in real spaces has not been verified. In this experiment, we created a small space where the observer could feel the color and scent simultaneously using the illumination color and observation box. This facilitated the verification of the cross-modal effects of the color and scent in real space. Another issue is the selection of a harmonious color for the scent. In previous studies, the color of the object itself was set as a harmony color (strawberry: red, lemon: yellow), and the cross-modal effect with other colors was often compared. With this method, it is difficult to state that the harmony between the color and scent can be sufficiently analyzed because the observer's prejudice is included, and the number of color samples to be compared is small.

To solve this problem, conditions were set for selecting harmonious colors in this experiment. The observers were asked whether the same “harmony” was the “smell harmony” or “image harmony.” The “smell harmony” refers to having the observer actually smell the scent and select a lighting color that they feel is in harmony. Conversely, “image harmony” refers to when the observer simply hears the name of the scent and is asked to select the lighting color to be recalled. By comparing the cross-modal effects of the two, we analyzed a wider range of colors and scent harmonies than in previous studies.

##### Preliminary Survey

The experiment was conducted with five Japanese university students. As a preliminary survey, the observers were asked to select a scent that they felt was “pleasant” and a scent that they felt was “unpleasant” from the five types of smell stimulations they created. This is to determine whether the activity of the orbitofrontal area is related to the feelings of the “pleasant” and “unpleasant” feelings and therefore, the effect is further promoted during the cross-modal of the color and scent. Table 1 lists the “pleasant” and “unpleasant” olfactory stimulations selected by the five observers.

**Table 1.** Observer-selected smell stimulation.

	Pleasant	Unpleasant
Observer 1	Lime	Cypress
Observer 2	Cypress	Mint
Observer 3	Vanilla	Cypress
Observer 4	Vanilla	Lime
Observer 5	Vanilla	Grapefruit

To select the five smell stimulations used in the experiment and the lighting color used to compare the cross-modal effects, we conducted three preliminary surveys with five observers. First, we asked the observers to select one of the five types of smell stimulations that they felt was the most “pleasant” and one that they felt was the most “unpleasant” and decided to use those two in a cross-modal experiment as smell stimulation. Subsequently, we evaluated the harmony between the fragrance and lighting colors when the observers actually smelled the fragrance. The observers observed 36 types of lighting colors while sniffing the fragrance and evaluated the degree of harmony for each lighting color on a 10-point scale. This process was performed for each of the “pleasant” and “unpleasant” fragrances selected by the observers. Finally, we determined the closeness between the colors that the observers recalled from the names of the fragrances and lighting colors. The observers were presented with 36 types of lighting colors for the names of the five essential oils, and the observers evaluated the closeness between the image color recalled from the names of the essential oils and lighting colors on a 10-point scale. We set the lighting colors that the observers felt were most in harmony as a / b and the lighting colors that they felt were most dissonant as c / d when they actually sniffed the fragrances with the “pleasant” and “unpleasant” fragrances. We also set the lighting colors that the observers felt most similar to the image color recalled from the name of the fragrance as a' / b' and the lighting colors that they felt most distant from each other as c' / b' with the “pleasant” and “unpleasant” fragrances. Eight lighting colors were used as the visual stimulation for the cross-modal experiments. According to these preliminary surveys, the smell and visual stimulation used in the cross-modal experiments differed among the five observers.

##### Experimental Method

**Figure 3.**
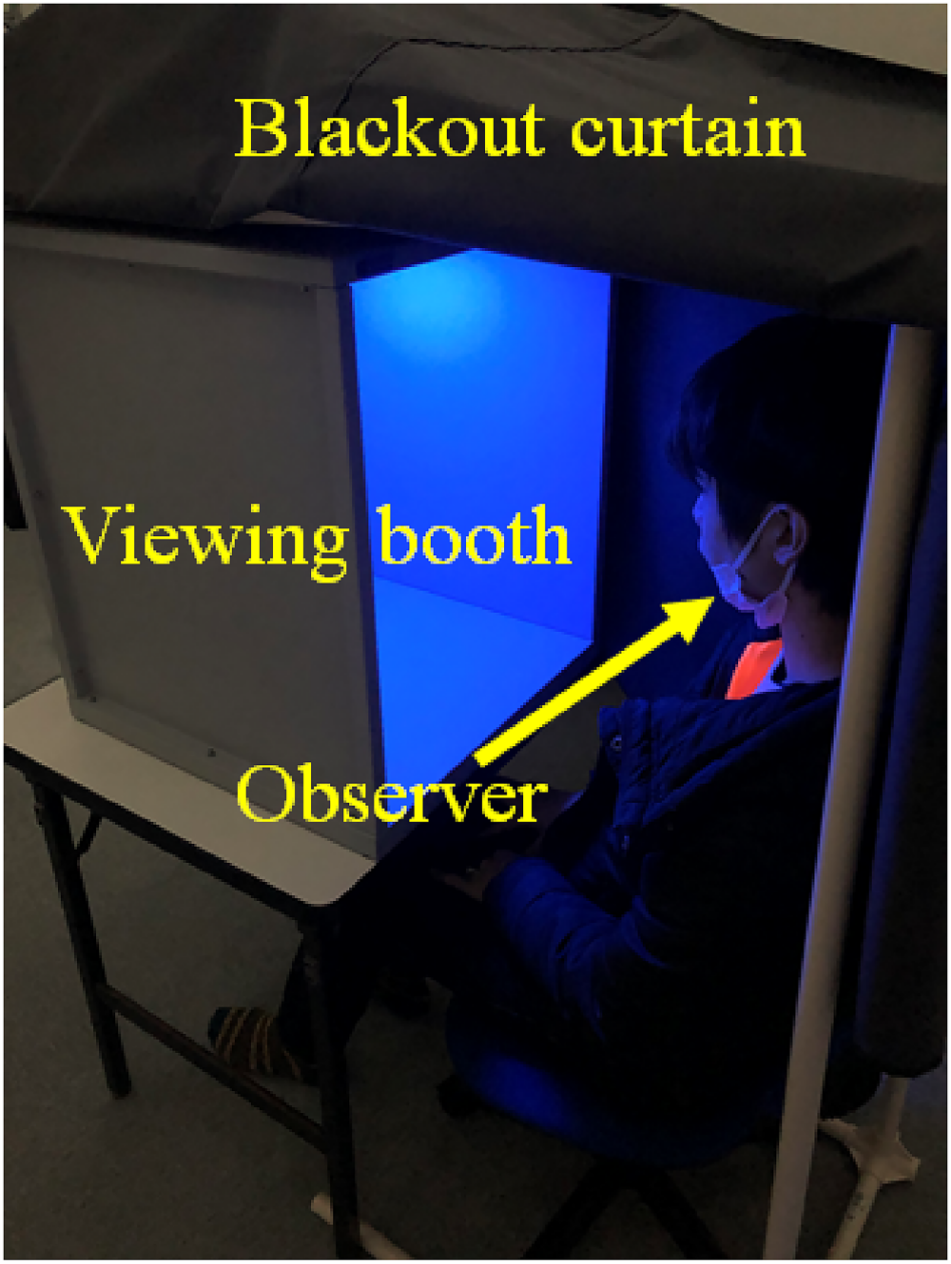
Experiment environment.

The experimental setup is illustrated in Figure 3. The LEDCube was installed on a 45 cm wide and 55 cm high, gray-walled viewing booth. The viewing booth with the LED cube was placed on a table and covered with a blackout curtain. The observers sat in chairs and were presented with the experimental stimuli. The temperature of the room where the experiments were conducted was fixed at 25 °C.

Three types of experiments were conducted by five observers. The breakdown of the three types of experiments is as follows: an experiment in which smell stimulation is presented alone, an experiment in which visual stimulation is presented alone, and a cross-modal experiment in which smell and visual stimulations are presented simultaneously. In this study, activity in the orbitofrontal cortex was recorded. Because this experiment involved a psychophysical test, the observers wearing the device were allowed to move slightly. NIRS (Hb133, Astem Co., Ltd.) was used in this study. NIRS has two channels, left and right, in the orbitofrontal portion of the brain; compared to fMRI, which can also measure brain activity, NIRS is more suitable for psychophysical experiments because it allows observers to move.

**Figure 4.**
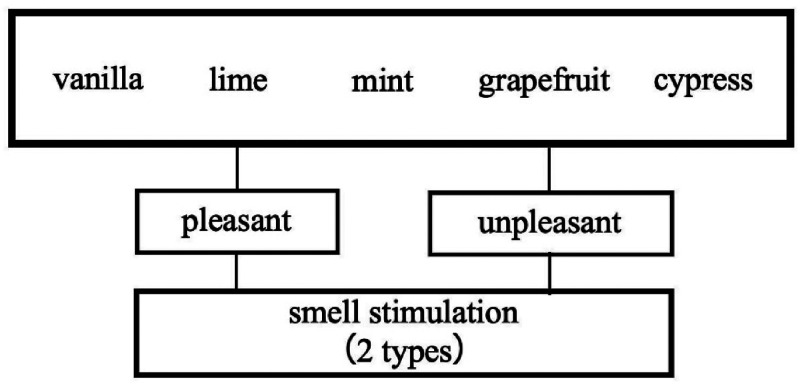
Sample selection method for smell stimulation experiments.

**Figure 5.**
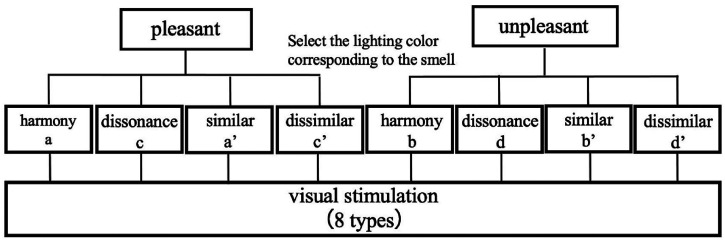
Sample selection method for visual stimulation experiments.

**Figure 6.**
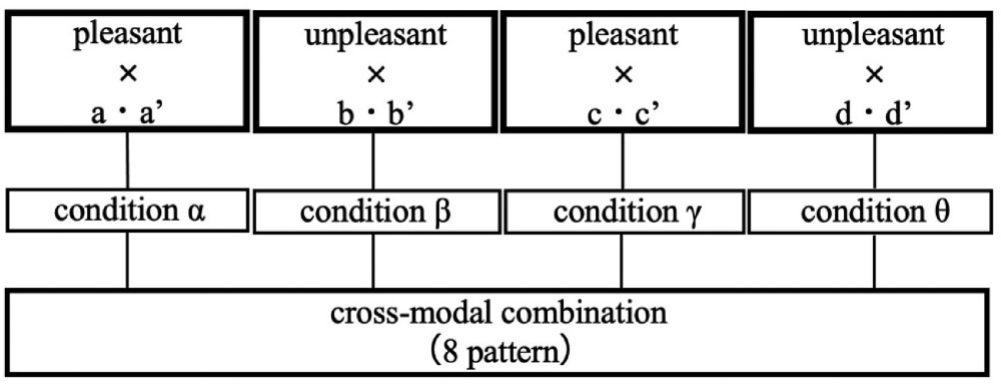
Combination of cross-modal experiments.

1. **Smell stimulation experiment**
  The method of the smell stimulation experiment is explained. The observer sat on a chair in the observation box and wore the NIRS. The illumination color in the observation box was set to white light (0.333, 0.333) as the reference light. The observer held a brown shaded bottle containing olfactory stimulation in his hand. At 60 s after the start of NIRS measurement, we gave a voice signal, and the observer confirmed it and immediately sniffed the smell stimulation for 30 s. The observer waited for 120 s. The smell stimulation varied between the observers. According to the preliminary survey, two patterns of the “pleasant” and “unpleasant” scents selected from five types were odor stimulations in this experiment. Two smell stimulation experiments were performed twice by the observers. There was also a 10-min break between each experiment. Figure 4 shows the selection method for the sample used in the smell stimulation experiment.
2. **Visual stimulation experiment**
  The method of the visual stimulation experiment is explained. As in the smell stimulation experiment, the observer wore the NIRS and sat in a chair, and the reference light was set for the lighting. Sixty seconds after the NIRS measurement started, the illumination of the observation box was changed from the reference light to the illumination color, which was a visual stimulation. Thirty seconds after the presentation of the visual stimulation, the light was returned to the reference light, and the observer was allowed to wait for 120 s. The visual stimulation varied among the observers. The illumination colors of the eight patterns (a, a', b, b', c, c', d, and d') selected in the preliminary survey were used as the visual stimuli in this experiment. Eight visual stimulation experiments were performed twice for each participant. There was also a 10-min break between each experiment. Figure 5 shows the selection of the sample used in the visual stimulation experiment.
3. **Cross-modal experiment**
  The method of the cross-modal experiment is explained. As in the smell stimulation experiment, the observer wore the NIRS and sat in a chair, and the reference light was set for the lighting. The observer then had olfactory stimulation of his hand in advance. At 60 s after the start of the NIRS measurement, the illumination of the observation box was changed from the reference light to the illumination color, which was a visual stimulation. After confirming this, the observer immediately sniffed the stimulation for 30 s. Thirty seconds after the lighting was changed, it was returned to the reference light again, and the observer finished sniffing the smell stimulation and waited for 120 s. In the cross-modal experiments, we conducted two patterns of lighting colors a and a' that were harmonious or similar to the pleasant fragrance (condition α), two patterns of lighting colors b and b' that were harmonious or similar to the unpleasant fragrance (condition β), two patterns of lighting colors c and c' that were dissonant or the images were separated by a pleasant fragrance (condition γ), and lighting colors d and d' that were dissonant or the images were separated by a pleasant fragrance (condition θ). Eight patterns of the cross-modal experiments were performed twice by the observers. There was also a 10-min break between each experiment. The details of the combination of the cross-modal experiments are shown in Figure 6.

### Experimental Results

**Figure 7.**
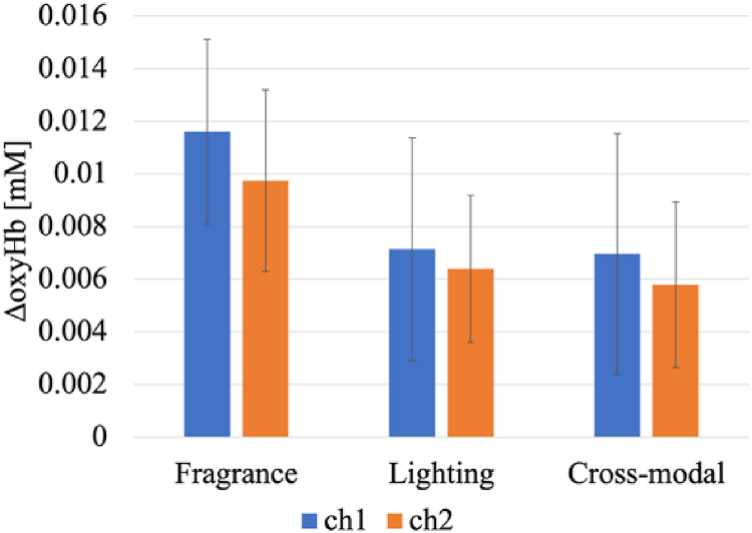
Amount of change in oxyHb by stimulation experiments. The error bars show the standard deviation of the two trials.

We calculated the average value of the two-day experiments with five observers and analyzed the results. The difference between the peak value until the end of the NIRS measurement and immediately before the presentation of the stimulation was defined as the amount of change in oxyHb. Figure 7 shows the calculated values for each stimulation experiment. The error bars show the standard deviation of the two trials. Comparing each stimulation experiment, it was found that the change in the oxyHb in the smell stimulation experiment was the largest. There was no significant difference between the visual stimulation and cross-modal experiments. We also observed that there was a difference in the amount of change in oxyHb between the channels. The NIRS used in these experiments could measure the orbitofrontal area on the two channels. Ch1 and Ch2 indicate the left and right side, respectively. Comparing the amount of change in oxyHb between the channels, it was found that the left orbitofrontal area changed significantly in all the stimulation experiments.

**Figure 8.**
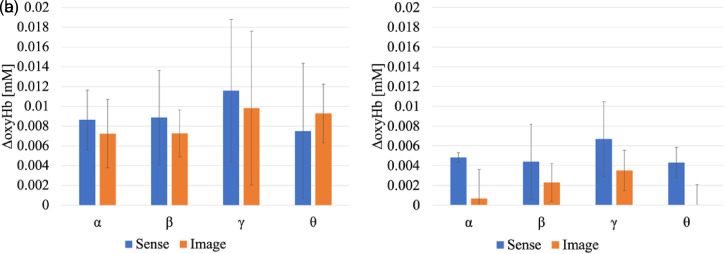
Amount of change in oxyHb by condition in cross-modal experiments. The error bars show the standard deviation of the two trials. (a) Peak value until the end of the measurement. (b) Peak value during stimulation.

Figure 8 shows the change in oxyHb for each condition in the cross-modal experiments. Figure 8(a) shows the difference between the peak value until the end of the NIRS measurement and immediately before the stimulation, and Figure 8(b) shows the difference between the peak value at 30 s during the stimulation and immediately before the stimulation. The error bars show the standard deviation of the two trials. At the time of the preliminary survey, the lighting colors that the observers actually sniffed the smell stimulation and judged to be in harmony or dissonance is expressed as “sense.” The lighting colors that the observers judged to be the closest to or far from the image color recalled from the name of the fragrance is expressed as “image.” From the results, it was found that the amount of change in sense was larger than that in the image under most conditions. Furthermore, it was found that the difference between the sense and image was more remarkable in Figure 8(b) than in Figure 8(a). From this, it can be inferred that the greater the harmony between the fragrance and lighting colors, the faster the reaction speed of the observers in the orbitofrontal area.

### Discussion

1. **Number of stimulation and orbitofrontal area reactions**
  The smell stimulation activated the orbitofrontal area of the observers more than the visual stimulation and cross-modal. It is probable that the orbitofrontal area was activated more because the observers carefully sniffed the essential oils that they were not accustomed to sniffing in white light, which was set as the reference light. We have previously compared experiments in which observers were made to wear eye masks and presented smell stimulation with complete obstruction of visual information and cross-modal experiments. At that time, the change in the oxyHb was larger in the cross-modal experiment. This is consistent with previous studies showing that the higher the number of stimulations taken by observers, the more active the orbitofrontal area (Danja et al., 2019).
2. **Left-right difference in orbitofrontal area activation**
  The left side of the orbitofrontal area was more activated than the right side in all of the smell, visual, and cross-modal stimulations. However, in previous studies that analyzed the response of the orbitofrontal area to the smell stimulation, opinions were divided because of laterality (Ishimaru et al., 2004; Osterbauer et al., 2005; Sara et al., 2019). Studies analyzing four brain regions that are the neural basis of “love” (Noriuchi et al., 2008) showed that the left side of the orbitofrontal area correlates with positive emotions and the right side with negative emotions. Because the smell stimulations used in these experiments were essential oils that produced relaxing effects, it is possible that positive emotions were promoted and the left side of the orbitofrontal area was more activated.
3. **The effect of harmony between scent and lighting color**
  The cross-modal (sense) with the lighting colors selected by the observers by sniffing the fragrance activates the orbitofrontal area more than the cross-modal (image) with the lighting colors selected by the observers by recalling the name of the fragrance. In other words, it can be observed that the cross-modal combination that the observers felt was in harmony with the senses was more pleasant than the prejudicial combination. Previous studies have confirmed that the greater the degree of harmony between the fragrance and color stimulation, such as color patches, the more active part of the brain is, including the orbitofrontal area (Danja et al., 2019; Osterbauer et al., 2005). This tendency is also obtained with lighting color, which is the visual stimulation of this study, and suggests that it can be applied to space design in real life. However, because sense is more active even under dissonant conditions, it can be inferred that the orbitofrontal area is activated even when it is more dissonant. Furthermore, it was found that the greater the degree of harmony, the faster the reaction speed in the orbitofrontal area. Studies have mentioned the effects of the harmony between the fragrance and color on the human task processing speed (Luisa et al., 2006). It can be deduced that this study successfully verified the relationship between the degree of harmony and human reaction speed from the viewpoint of brain waves, which is a physiological aspect.

#### Verification Experiment

The experiments have demonstrated that harmony in color and scent in a lighting environment affect the activity of the orbitofrontal area. In the above, it was stated that the combination of “sensually selected” colors and scents promotes “pleasant” emotions more than the “image-selected” combination. However, it cannot be inferred that the “degree of harmony” between the color and scent clearly influence emotion. In this additional experiment, we evaluated the effects of color and scent harmony on emotions.

In the preliminary survey of the previous experiment, the observers evaluated the smell harmony of all 36 types of lighting colors for the “pleasant” scent on a 10-point scale. From this, “10 lighting colors,” “7 lighting colors,” and “5 lighting colors” were extracted, and a cross-modal experiment with the scent of “pleasant” was conducted. If the magnitude of the harmony between the color and scent influences the feeling of “pleasure,” the amount of change in oxyHb is in the order of 10 lighting colors → 7 lighting colors → 5 lighting colors. The experiment was performed by three observers of the previous experiment.

**Figure 9.**
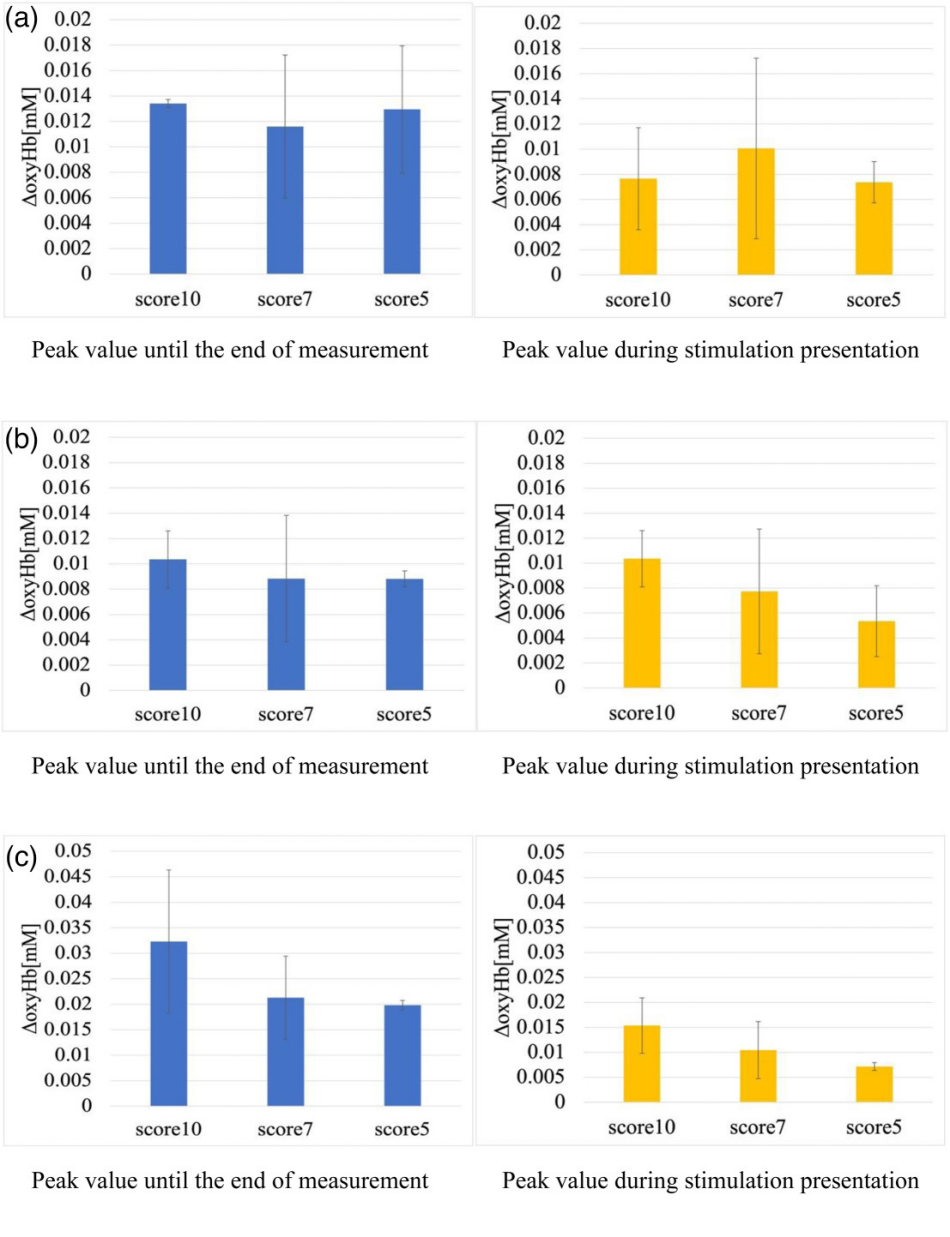
Amount of change in oxyHb by degree of harmony between color and scent. The error bars show the standard deviation of the two trials. (a) Observer 1, (b) Observer 2, (c) Observer 3.

The average value from the two experiments was calculated for each of the three observers and the analysis was performed by focusing on the amount of change in oxyHb. Figure 9 shows the difference between the peak value until the end of measurement and immediately before the stimulation in NIRS, and the difference between the peak value during the stimulation and immediately before the stimulation. Error bars in the graph represent the standard deviations of the two trials.

For observer 1, no correlation was observed between the degree of harmony and change in the oxyHb levels. However, for the other two observers, the amount of change in oxyHb increased in the order of harmony. Furthermore, the peak value during the presentation of the stimulation was also correlated with the magnitude of the degree of harmony. From this result, it was confirmed that the degree of harmony between the color and scent has a correlation with the amount of change in oxyHb even in a lighting environment and influences the feeling of “pleasure.” The peak values during the presentation of the stimulation also suggest that the magnitude of color and scent harmony affected the human reaction rate.

#### Conclusion

In this study, we analyzed the relationship between the color and scent harmony in a lighting environment from the viewpoint of brain activity, which is a physiological index. Among the brain activities, we focused on the orbitofrontal area, which is said to correlate with “pleasant” emotions. NIRS, which can tolerate relative body movement and has been confirmed to be reproducible in conventional studies, was used as a device for measuring the activity of the prefrontal cortex of the orbit.

Using essential oils for olfactory stimulation and a spectral tunable lighting device for visual stimulation, a cross-modal experiment was conducted in which the essential oils were presented at the same time as the experiments in which they were presented independently. For olfactory stimulation, we selected five types of aromas that were familiar to the Japanese observers. For the illumination color, which is a visual stimulation, we referred to the vivid tone of the PCCS. Twelve types of hues were further subdivided into three saturations, and the harmony between the color and scent was evaluated. In the experiment, the observers were asked to select two types of olfactory stimulation, “pleasant” and “unpleasant,” by a preliminary survey. Additionally, a method different from conventional research was used to select the harmony color. By letting the observer select a lighting color that is “sense” harmonized or dissonant with the smell stimulation and a lighting color that is “image” similar or dissimilar, a wider range of colors than in previous studies can be achieved.

We analyzed three types of experiments focusing on the amount of change in oxyHb in the orbitofrontal area. The smell stimulation experiment was significantly different from the visual stimulation and cross-modal experiments. This was inferred to be due to the fact that the observer was carefully sniffing the essential oil, which is a strong stimulation that he or she is not normally familiar with, because the smell stimulation experiment was conducted first among the three experiments. Furthermore, in all the experiments, the left orbitofrontal area was more active than the right orbitofrontal area. In the previous study, there was disagreement on the difference in the activation in the orbitofrontal area, including verification by fMRI; however, in this study, the relaxing effect of essential oil affects the feeling of “pleasure,” and it is a positive feeling. The correlated left orbitofrontal area was considered activated. It was also confirmed that the smell harmony of the color and scent activated the orbitofrontal area and affected the reaction rate compared with the case selected in the image.

In additional experiments, we examined the relationship between the color and scent harmony and activity in the orbitofrontal area. When performed by three observers, two showed a correlation between the degree of harmony and the amount of change in oxyHb. These results suggest that the harmony between the color and scent affects human emotions, even in a lighting environment.

By further increasing the number of experiments and improving the accuracy, it is desirable to apply it to real-space design, considering the smell harmony of the color and scent.
